# Longitudinal analysis of HIV risk behaviour patterns and their predictors among public primary care patients with tuberculosis in South Africa

**DOI:** 10.1080/17290376.2018.1433057

**Published:** 2018-02-06

**Authors:** Karl Peltzer

**Affiliations:** ^a^ HIV/AIDS/STIs and TB (HAST), Human Sciences Research Council, Pretoria, South Africa; ^b^ Department of Research & Innovation, University of Limpopo, Turfloop Campus, Sovenga, South Africa

**Keywords:** sexual risk behaviour, TB patients, health status, mental health, longitudinal study, South Africa

## Abstract

The goal of this study was to identify various HIV risk behaviours among tuberculosis (TB) patients in a longitudinal study design in South Africa. In 42 public primary healthcare facilities in three districts in three provinces, adult new TB and TB retreatment patients with hazardous or harmful alcohol use were interviewed within 1 month of initiation of anti-TB treatment and were followed up at 6 months. The total sample with a complete 6-month follow-up assessment was 853. At the follow-up assessment, several HIV risk behaviours significantly reduced from baseline to follow-up. In multivariate Generalized Estimating Equations logistic regression analyses, high poverty (odds ratio (OR): 2.68, 95% confidence interval (CI): 1.56–4.62), Posttraumatic Stress Disorder (PTSD) symptoms (OR = 1.55, 95% CI = 1.03–2.36), and sexual partner on antiretroviral therapy (ART) (OR = 1.84, 95% CI = 1.09–3.10) were associated with a higher odds, and excellent/very good perceived health status (OR: 0.61, 95% CI: 0.37–0.98), severe psychological stress (OR = 0.51, 95% CI = 0.34–0.77), and HIV non-disclosure to most recent sexual partner (OR = 0.40, 95% CI = 0.25–0.65) were associated with a lower odds of inconsistent condom use. Being HIV positive (OR = 4.18, 95% CI = 2.68–6.53) and excellent/very subjective health status (OR = 2.98, 95% CI = 1.73–5.13) were associated with a higher odds, and having PTSD symptoms (OR = 0.60, 95% CI = 0.36–0.99), being on ART (OR = 0.48, 95% CI = 0.25–0.95), having a sexual partner on ART (OR = 0.41, 95% CI = 0.18–0.96), and HIV status non-disclosure (OR = 0.25, 95% CI = 0.15–0.41) were associated with a lower odds of having sex with an HIV-positive or HIV status unknown person. High poverty index (OR = 1.97, 95% CI = 1.19–3.25) and having a sexual partner on ART (OR = 4.37, 95% CI = 1.82–10.48) were associated with a higher odds, and having a partner with HIV-negative status (OR = 0.29, 95% CI = 0.16–0.51) and inconsistent condom use (OR = 0.39, 95% CI = 0.24–0.64) were associated with a lower odds of HIV status non-disclosure at last sex. The study found that among TB patients with problem drinking over a 6-month TB treatment period, the frequency of some HIV risk behaviours (inconsistent condom use) declined (OR = 0.64, 95% CI = 0.41–0.98), but also persisted at a high-level calling for a strengthening and integration of HIV prevention into TB management.

## Introduction

According to the latest global tuberculosis (TB) report (WHO, 2015), South Africa has the highest (696) per 100,000 TB prevalence, and the HIV prevalence incident TB cases is 61% (WHO, 2015). The high rate of HIV co-infections among TB patients in South Africa emphasizes the urgent need for HIV reduction interventions in this subpopulation. Risky sexual behaviour such as lack of condom use and multiple sexual partners (Degefa, 2006; Kigosi, Heunis, Chikobvu, Wouters, & van den Berg, 2011; Mankatittham et al., 2009; Matseke et al., 2012; Talbot et al., 2002; Zacharia, Spielmann, Harries, & Salaniponi, 2003) have been found among individuals having TB and TB-HIV co-infected. In a large study in South Africa, 63% of TB patients had inconsistently used condoms in the past 3 months (Matseke et al., 2012); in TB patients in Botswana, the majority had inconsistently used a condom (Talbot et al., 2002); in adult TB patients in Ethiopia, 78% had not used a condom (Degefa, 2006); and among HIV-infected TB patients in Thailand, 42% reported never using condoms at all (Mankatittham et al., 2009).

In studies with HIV risk groups (key populations such as sex workers, intravenous drug users and men who have sex with men, and HIV-infected and patients who commenced antiretroviral therapy (ART)) other than TB patients, generally a decline or reduction of sexual HIV risk behaviour was observed over time (Holtz et al., 2015; McClelland et al., 2010; Ndziessi et al., 2013; Peltzer & Ramlagan, 2010; Sou et al., 2015).

Before implementing suitable risk reduction interventions, it is vital to identify factors that may influence risk behaviour. In HIV risk groups (key populations such as sex workers and men who have sex with men, and HIV-infected and patients who commenced on ART), other than TB patients, the following factors were identified for HIV sexual risk behaviour: difficulty accessing condoms (Sou et al., 2015), being HIV negative (Holtz et al., 2015), poor mental health (Bouhnik et al., 2002; Holtz et al., 2015; Theall, Clark, Powell, Smith, & Kissinger, 2007), binge drinking (Holtz et al., 2015; Theall et al., 2007), know or think current partner is HIV positive (Theall et al., 2007), partner HIV positive or of unknown status (Bouhnik et al., 2002), being on ART (Bouhnik et al., 2002; Theall et al., 2007), and non-disclosure of HIV status (Venkatesh et al., 2010). Longitudinal studies of sexual risk behaviour in TB patients are lacking. The goal of this study was to identify various HIV risk behaviours and their predictors among TB patients in a longitudinal study design.

## Methods

### Study design

This is a longitudinal observational study of TB patients with hazardous or harmful drinking problems in public primary care clinics in South Africa.

### Sample and procedure

In 42 public primary healthcare facilities in three districts in South Africa, all adult new TB and TB retreatment patients with hazardous or harmful alcohol use were consecutively interviewed within 1 month of initiation of anti-TB treatment over a period of 6 months and were followed up at 6 months of treatment. A research assistant obtained informed consent from these patients attending the primary care facility to participate in the interview. Questionnaires were translated and translated back into the major languages of the study participants (Afrikaans, Tswana, Xhosa, and Zulu) (Peltzer, Naidoo, Matseke, & Zuma, 2011). More details of the study methodology are described elsewhere (Peltzer et al., 2013).

Ethical approval was obtained from the Human Sciences Research Council Research Ethics Committee (Protocol REC No.1/16/02/11) and the Department of Health in South Africa. In this study sample, only TB patients who completed the 6-month follow-up were included (Peltzer et al., 2013).

### Measures

*Health status variables*: TB treatment status and HIV status and treatment status were assessed by self-report and from medical information. *Perceived general health*: Participants were asked: In general, would you say your health is: excellent, very good, good, fair or poor? This measure was categorized based on participant response (1 = fair/poor, 2 = good, 3 = excellent/very good) (Üstün, Kostanjsek, Chatterji, & Rehm, 2010).

*Condom use* was assessed with the question, ‘How frequently did you use condoms when you had sex with your most recent sexual partner in the past three months? Response options were 1 = Always, 2 = Frequently, 3 = Sometimes, and 4 = Never. Inconsistent condom use was defined as not always using a condom with the most recent sexual partner in the past 3 months. Sexual partner characteristics were asked with the questions, “What is the HIV status of your most recent sexual partner?’ (Response options: HIV positive, HIV negative, Do not know), ‘Have you disclosed your HIV status to this partner?’ (Response options: yes or no), and ‘Is your sexual partner on ART’ (Response options: yes or no) (Peltzer & Ramlagan, 2010; Schroder, Carey, & Vanable, 2003).

The *Kessler Psychological Distress Scale* (K-10) was used to measure global psychological distress, including significant pathology which does not meet formal criteria for a psychiatric illness (Kessler et al., 2002). This scale serves to identify individuals who are likely to meet formal definitions for anxiety and/or depressive disorders, as well as to identify individuals with subclinical illness who may not meet formal definitions for a specific disorder (Kessler et al., 2002). The K-10 scale was examined using a cut-off of 28, indicating severe psychological distress, as found in a clinical validation study in South Africa (Spies et al., 2009). The internal reliability coefficient for the K-10 in this study was alpha = 0.92 at Time 1 and 0.87 at Time 2.

*Primary Care PTSD Screen* (PC-PTSD): The four items of the PC-PTSD (Prins et al., 2003) correspond to the four factors (i.e. re-experiencing, avoidance, hyperarousal, and numbing) found to be specific to the PTSD conduct. The first item included, ‘In your life have you ever had any experience that was so frightening, horrible, or upsetting that in the past month you have had nightmares about it or thought about it when you did not want to?’ Items are endorsed using a yes/no format. The PC-PTSD is a four-item screen that was designed for use in primary care and other medical settings. A cut-off of 2 was used for PTSD-positive screening (Prins et al., 2003). The Cronbach alpha for the PC-PTSD in this study was 0.89 at Time 1 and 0.92 at Time 2.

*Socio-economic characteristics* included age, gender, and educational level, and *Poverty* was assessed by five items asking about the availability or non-availability of shelter, fuel or electricity, clean water, food, and cash income in the past week (Peltzer et al., 2011). An example item: ‘In the past week, how often have you gone without enough food to eat?’ Response options ranged from 1 = not one day to 4 = every day of the week. The total score ranged from 5 to 20: 5 = being low on poverty, 6–12 = medium level of poverty, and 13–20 = high levels of poverty. The Cronbach alpha for the poverty index in this study was 0.89 at Time 1 and 0.96 at Time 2.

### Data analysis

Data were analysed using the Statistical Package for the Social Sciences (SPSS) for Windows software application programme version 22. Of the 1196 TB patients enrolled in the study, the prospective longitudinal analysis was restricted to 853 (71.3%) of the TB patients. Descriptive statistics were done at baseline and stratified by follow-up results in health and sexual risk variables. The differences between baseline and follow-up assessments were assessed using McNemar’s test. Correlates of three sexual risk behaviours over the 6-month follow-up period were examined longitudinally, using multivariable logistic regression using Generalized Estimating Equations (GEE). GEE takes care of missing data utilizing the GEE estimating equation that substitutes data from non-missing pairs into the estimators of the correlation matrix (Argento et al., 2014). Socio-demographic, TB treatment status, and HIV status were treated as fixed covariates, while all other health and sexual risk variables were treated as time-updated covariates. A *P*-value below .05 was regarded as statistically significant.

## Results

### Sample characteristics

The total sample with a complete 6-month follow-up assessment was 853 and they were eligible for this analysis. The mean age was 36.8 years (SD = 11.0). The majority were men (71.9%), 73.2% had Grade 8 or more education, 33.7% had a high poverty index, 21.3% were TB retreatment patients, and 54.9% were TB-HIV co-infected (see Table 1).

**Table 1.** Socio-demographic and health status sample characteristics of TB patients with problem drinking at baseline.

Variables	Baseline (Time 1) (*N* = 853)
	*N* (%)
Socio-economic factors	
Age in years	
18–34	266 (44.6)
35–44	159 (26.7)
45 or older	171 (28.7)
Gender	
Male	432 (71.9)
Female	169 (28.1)
Education	
Grade 7 or less	164 (26.8)
Grade 8–11	354 (57.7)
Grade 12 or more	95 (15.5)
Poverty index	
Low	223 (37.2)
Medium	174 (29.0)
High	202 (33.7)
Health status	
TB treatment status	
New treatment	648 (78.7)
Retreatment	175 (21.3)
HIV positive	459 (54.9)

At the follow-up assessment, subjective health status had much improved and mental morbidity (psychological distress and PTSD symptoms) significantly reduced at 6 months TB treatment. Several HIV risk behaviours (inconsistent condom use, most recent sexual partner with HIV positive or unknown status, HIV non-disclosure and not being on ART) significantly reduced from baseline to follow-up (see Table 2).

**Table 2.** Health and sexual risk variables characteristics of TB patients at Time 1 and Time 2.

Variables	Baseline	Month 6	*P*
	%	%	
Health status			
Perceived health status			
Fair/poor	41.9	19.9	<.001
Good	36.2	31.8	
Excellent/very good	21.9	48.3	
Mental health			
Severe psychological distress	33.9	21.8	.001
PTSD symptoms	30.0	9.4	<.001
Sexual risk variables			
ART status			
On ART	20.7	31.8	<.001
Sexual partner on ART	11.0	12.9	<.001
Sexual partner and behaviour			
HIV non-disclosure to most recent sexual partner	42.1	37.0	<.001
Most recent sexual partner with HIV positive or unknown status	78.2	67.3	<.001
Inconsistent condom use	70.4	51.2	.033

### Longitudinal analyses of HIV risk variables

In multivariate GEE logistic regression analyses, medium (odds ratio (OR): 1.47, 95% confidence interval (CI): 1.01–2.15) and high poverty (OR: 2.68, 95% CI: 1.56-4.62), PTSD symptoms (OR = 1.55, 95% CI = 1.03–2.36), and sexual partner on ART (OR = 1.84, 95% CI = 1.09–3.10) were associated with a higher odds of inconsistent condom use; excellent/very good perceived health status (OR = 0.61, 95% CI = 0.37–0.98), severe psychological stress (OR = 0.51, 95% CI = 0.34–0.77), and HIV non-disclosure to most recent sexual partner (OR = 0.40, 95% CI = 0.25–0.65) were associated with a lower odds of inconsistent condom use. Furthermore, being HIV positive (OR = 4.18, 95% CI = 2.68–6.53) and excellent/very subjective health status (OR = 2.98, 95% CI = 1.73–5.13) were associated with a higher odds of having sex with an HIV-positive or HIV status unknown person; having PTSD symptoms (OR = 0.60, 95% CI = 0.36–0.99), being on ART (OR = 0.48, 95% CI = 0.25-0.95), having a sexual partner on ART (OR = 0.41, 95% CI = 0.18–0.96), and HIV status non-disclosure to most recent sexual partner (OR = 0.25, 95% CI = 0.15–0.41) were associated with a lower odds of having sex with an HIV-positive or HIV status unknown person. High poverty index (OR = 1.97, 95% CI = 1.19–3.25) and having a sexual partner on ART (OR = 4.37, 95% CI = 1.82–10.48) were associated with a higher odds of HIV status non-disclosure at last sex; having a partner with HIV-negative status (OR = 0.29, 95% CI = 0.16–0.51) and inconsistent condom use (OR = 0.39, 95% CI = 0.24–0.64) were associated with a lower odds of HIV status non-disclosure at last sex. Furthermore, compared with the baseline assessment, the frequency of inconsistent condom use declined (OR = 0.64, 95% CI = 0.41–0.98) at the follow-up assessment but remained unchanged for the other two HIV risk behaviours (see Table 3).

**Table 3.** Multivariate GEE logistic regression analyses of predictors for three HIV risk behaviours.

Variables	Inconsistent condom use in past 3 months	Sex with partner with HIV positive or unknown status	HIV status non-disclosure at last sex
	AOR (95% CI)	AOR (95% CI)	AOR (95% CI)
Socio-economic factors			
Age in years			
18–34	1.00	1.00	1.00
35–44	1.20 (0.75–1.94)	1.05 (0.62–1.78)	0.83 (0.55–1.27)
45 or more	0.71 (0.40–1.25)	1.56 (0.91–2.67)	1.17 (0.76–1.81)
Gender			
Male	1.00	1.00	1.00
Female	0.66 (0.44–100)	1.01 (0.63–1.60)	1.24 (0.83–1.86)
Education			
Grade 7 or less	1.00	1.00	1.00
Grade 8–11	0.85 (0.51–1.40)	0.92 (0.50–1.70)	0.78 (0.52–1.18)
Grade 12 or more	0.65 (0.35–1.20)	0.87 (0.41–1.91)	0.85 (0.51–1.42)
Poverty index			
Low	1.00	1.00	1.00
Medium	1.47 (1.01– 2.15)*	1.15 (0.72–1.86)	0.86 (0.58–1.27)
High	2.68 (1.56–4.62)***	0.86 (0.49–1.51)	1.97 (1.19–3.25)**
Health status			
HIV positive (base = HIV negative or unknown status)	0.83 (0.54–1.26)	4.18 (2.68–6.53)***	0.87 (0.58–1.31)
TB treatment status			
New treatment	1.00	1.00	1.00
Retreatment	1.18 (0.75–1.87)	1.14 (0.64–2.04)	0.76 (0.48–1.20)
Perceived health status			
Fair/poor	1.00	1.00	1.00
Good	0.97 (0.66–1.45)	1.87 (1.13–3.09)*	1.06 (0.70–1.59)
Excellent/very good	0.61 (0.37–0.98)*	2.98 (1.73–5.13)***	0.92 (0.57–1.47)
Severe psychological distress	0.51 (0.34–0.77)***	0.98 (0.62–1.55)	1.06 (0.71–1.58)
PTSD symptoms	1.55 (1.03–2.36)*	0.60 (0.36–0.99)*	1.41 (0.93–2.14)
Sexual risk variables			
On ART	1.21 (0.79–1.87)	0.48 (0.25–0.95)*	1.31 (0.72–2.38)
Sexual partner on ART	1.84 (1.09–3.10)*	0.41 (0.18–0.96)*	4.37 (1.82–10.48)***
HIV non-disclosure to most recent partner	0.40 (0.25–0.65)***	0.25 (0.15–0.41)***	N/A
Most recent sexual partner with HIV-positive or unknown status	0.87 (0.54–1.41)	N/A	0.29 (0.16–0.51)***
Inconsistent condom use	N/A	0.87 (0.53–1.43)	0.39 (0.24–0.64)***
Time of assessment			
Time 1	1.00	1.00	1.00
Time 2	0.64 (0.41–0.98)*	1.01 (0.63–1.63)	0.81 (0.51–1.27)

***P < .001; **P < .01; *P < .05.

AOR: adjusted odds ratio; CI: confidence interval.

## Discussion

The study found that among TB patients with problem drinking over a 6-month TB treatment period, the frequency of some HIV risk behaviours (inconsistent condom use) declined. This result is in agreement with several studies with HIV risk groups (sex workers, intravenous drug users and men who have sex with men, and HIV-infected and patients who commenced ART) other than TB patients (Holtz et al., 2015; McClelland et al., 2010; Ndziessi et al., 2013; Peltzer & Ramlagan, 2010; Sou et al., 2015). Some of the reduction of sexual risk behaviour over time may be attributed to HIV healthcare interventions in the TB clinics. However, TB patients still maintained a high proportion of HIV risk behaviours (51.2% inconsistent condom use, 67.3% having sex with HIV-positive or unknown status, 37% HIV non-disclosure to most recent sexual partner), which conforms with previous cross-sectional studies with TB patients in South Africa, Botswana, Ethiopia, and Thailand (Degefa, 2006; Mankatittham et al., 2009; Matseke et al., 2012; Talbot et al., 2002). This result calls for strengthening and integration of HIV prevention interventions into TB programmes. Furthermore, efforts should also be made for a follow-up couple counselling to overcome barriers of disclosure. Although condoms are freely available in public health facilities in South Africa and are widely promoted in mass media and other HIV prevention campaigns, more needs to be done to promote consistent condom use (Peltzer, 2013).

Furthermore, the results of the study suggest that people who were living in poverty were more likely to report inconsistent condom use and HIV non-disclosure compared to their respective counterparts. Similar to the results suggested in this study, poverty was found to be associated with risky sexual behaviour (multiple partnerships) in South Africa (Booysen, 2004), and food insufficiency was identified as an important risk factor for increased sexual risk-taking (inconsistent condom use) in Botswana and Swaziland (Weiser et al., 2007). Although having PTSD symptoms were found to be associated with inconsistent condom use in this study, as in previous studies, poor mental health (Bouhnik et al., 2002; Holtz et al., 2015; Theall et al., 2007) was associated with HIV sexual risk behaviour and severe psychological distress was inversely correlated with inconsistent condom use.

Being HIV positive was highly associated with recent sex with an HIV-positive or an unknown status partner. It is possible that a high proportion of participants engaged in serocordant sex. Unlike in a previous study (Bouhnik et al., 2002), this study did not find any association between having an HIV-positive or of unknown status partner and sexual risk behaviour. Further studies investigating HIV sexual risk behaviours longitudinally among HIV-positive TB patients are needed.

Being on ART was, unlike in previous studies (Bouhnik et al., 2002; Theall et al., 2007), inversely associated with HIV risk behaviour in this study. However, with a sexual partner on ART, the odds increased for inconsistent condom use and for HIV status non-disclosure. It is possible that TB patients believe that a sexual partner on ART is less or not infectious for HIV transmission, and therefore engage in unprotected sex.

Previous studies found that non-disclosure of HIV status (e.g. Venkatesh et al., 2010) was associated with HIV sexual risk behaviour, while in this study, HIV status disclosure increased the odds of inconsistent condom use. Furthermore, consistent condom use and having had sex with an HIV-negative person were associated with HIV status non-disclosure.

### Study limitations

Caution should be taken when interpreting the results of this study because of certain limitations. Generalizability of our findings is limited to TB patients with alcohol problems during anti-TB treatment in public primary care centres in South Africa. A further limitation was that most variables were assessed by self-report and socially desirable responses may have been given or a recall bias may have occurred. Furthermore, the loss to follow-up of TB patients could have introduced a bias (Peltzer et al., 2013). Moreover, certain concepts like having multiple sexual partners were not assessed and should be included in future studies. Since all TB patients had been problem drinkers, the study could not compare problem and non-problem drinkers in relation to sexual risk behaviour, as a significant association had been found in previous studies (Holtz et al., 2015; Theall et al., 2007).

## Conclusion

The study found that among TB patients with problem drinking over a 6-month TB treatment period, the frequency of some HIV risk behaviours (inconsistent condom use) declined. However, there was also a persistence of various sexual risk behaviours, calling for a strengthening and integration of HIV prevention into TB management. Several predictors associated with sexual risk behaviours were identified, which can help guide interventions.
